# Parkinson Symptoms and Health Related Quality of Life as Predictors of Costs: A Longitudinal Observational Study with Linear Mixed Model Analysis

**DOI:** 10.1371/journal.pone.0145310

**Published:** 2015-12-23

**Authors:** Pablo Martinez-Martín, Carmen Rodriguez-Blazquez, Silvia Paz, Maria João Forjaz, Belén Frades-Payo, Esther Cubo, Jesús de Pedro-Cuesta, Luis Lizán, ELEP Group

**Affiliations:** 1 National Center of Epidemiology and CIBERNED, Carlos III Institute of Health, Madrid, Spain; 2 Outcomes’ 10, Jaume I University, Castellon de la Plana, Castellon, Spain; 3 National School of Public Health and REDISSEC, Carlos III Institute of Health, Madrid, Spain; 4 Research Unit, Alzheimer Center Reina Sofia Foundation, Carlos III Institute of Health, Madrid, Spain; 5 Complejo Asistencial Universitario de Burgos, Burgos, Spain; Instituto Cajal-CSIC, SPAIN

## Abstract

**Objective:**

To estimate the magnitude in which Parkinson’s disease (PD) symptoms and health- related quality of life (HRQoL) determined PD costs over a 4-year period.

**Materials and Methods:**

Data collected during 3-month, each year, for 4 years, from the ELEP study, included sociodemographic, clinical and use of resources information. Costs were calculated yearly, as mean 3-month costs/patient and updated to Spanish €, 2012. Mixed linear models were performed to analyze total, direct and indirect costs based on symptoms and HRQoL.

**Results:**

One-hundred and seventy four patients were included. Mean (SD) age: 63 (11) years, mean (SD) disease duration: 8 (6) years. Ninety-three percent were HY I, II or III (mild or moderate disease). Forty-nine percent remained in the same stage during the study period. Clinical evaluation and HRQoL scales showed relatively slight changes over time, demonstrating a stable group overall. Mean (SD) PD total costs augmented 92.5%, from €2,082.17 (€2,889.86) in year 1 to €4,008.6 (€7,757.35) in year 4. Total, direct and indirect cost incremented 45.96%, 35.63%, and 69.69% for mild disease, respectively, whereas increased 166.52% for total, 55.68% for direct and 347.85% for indirect cost in patients with moderate PD. For severe patients, cost remained almost the same throughout the study. For each additional point in the SCOPA-Motor scale total costs increased €75.72 (p = 0.0174); for each additional point on SCOPA-Motor and the SCOPA-COG, direct costs incremented €49.21 (p = 0.0094) and €44.81 (p = 0.0404), respectively; and for each extra point on the pain scale, indirect costs increased €16.31 (p = 0.0228).

**Conclusions:**

PD is an expensive disease in Spain. Disease progression and severity as well as motor and cognitive dysfunctions are major drivers of costs increments. Therapeutic measures aimed at controlling progression and symptoms could help contain disease expenses.

## Introduction

Parkinson’s disease (PD) is the second most common neurodegenerative disorder after Alzheimer’s disease and its prevalence will continue to grow as the population ages. More than one million people are diagnosed with PD in Europe and up to 5 million worldwide [1]. The prevalence increases with age, affecting approximately 1% of those aged 60 years or older, 4% or more of those aged 80 years or older and approximately 5.2% of those in nursing homes [1]. Given the growing elderly population in Europe, this number is forecast to double by 2030 [2]. The last studies performed in Spain have calculated a prevalence of PD between 161-270/100,000 inhabitants, giving a total of 160,000 patients [3].

PD symptoms can be classified as motor or non-motor. The first includes tremor, rigidity, bradykinesia and postural instability. Non-motor symptoms are varied, such as loss of taste and sense of smell, sleep disturbances, gastrointestinal complications, constipation, swallowing problems, anxiety, pain, fatigue, depression, sexual dysfunction, hallucinations and psychosis, impulse control disorders, cognitive impairment and dementia [4].

The economic impact of the disease is enormous. In 2011, the annual European cost was estimated at €13.9 billion, and it is known to increase with the progression of the disease [5]. In Germany, disease total cost in 2010 varied from €18,660 for mild disease to €31,660 for moderate and severe PD [6]. UK 2011 figures supports this view, indicating that moderate and severe disease can cost up to €72,277 per person [7]. Total costs can be divided in direct, which include consultations, hospital admissions, pharmacologic treatment, among others; indirect costs, including early retirement and loss of productivity due to disability; and intangible costs, including dependency, physiological effects, pain, etc. [8, 9].

Both motor- and non-motor symptoms can result on hospitalizations, which causes an increase of healthcare resources utilization and a significant escalation of economic burden, as well as augmentation of indirect costs because of its relation with patient’s disability, which can affect work productivity of both patient and caregivers. Moreover, non-motor symptoms impair patients’ quality of life, which relates to intangible costs [10].

Advanced PD is associated with increased cost and decreased health related quality of life (HRQoL) [9]. Slowing disease progression through specific neuro-protective mechanisms, such as effective drug treatments in combinations with efficient and safe therapeutic interventions may result in less need for care, which in turn can translate into reduced associated costs and improved HRQoL for patients and caregivers [11, 12].

Several publications have analyzed the economic burden of PD in Europe [5, 6, 7] but none has estimated longitudinal costs variations in relation to disease severity, symptoms or HRQoL, information much needed for reckoning how medical spending occurs throughout the natural history of the disease. This study aim was to estimate the magnitude in which disease symptoms and HRQoL determined the direct and indirect costs of Parkinson over a 4-year period.

## Materials and Methods

### Study Design

This costs study is part of the observational, cross sectional, multicenter, nationwide, longitudinal ELEP study (Longitudinal Parkinson's Disease Patient Study -Estudio Longitudinal de Pacientes con Enfermedad de Parkinson). ELEP aimed at describing the course of PD motor and non-motor symptoms, its impact on disability and HRQoL impairment, long-term effect of available therapeutics and the impact of the disease among caregivers [13].

Participants were included in accordance to the criteria followed by the ELEP study, previously published [13]. Briefly, patients were required to be at least 30 years old, diagnosed by a neurologist in accordance with the United Kingdom PD Society Brain Bank (UKPDSBB) criteria [14] and provided written consent for their participation. The inclusion of participants was performed in groups of 8, distributed according to sex (female or male), age of PD symptoms onset (younger or older than 60 years old) and disease duration (shorter or longer than 5 years). Patients were excluded if they lack of a stable caregiver or because of any condition that prevented a thorough disease evaluation.

### Ethical Aspects

Both patients and caregivers signed an informed consent to participate in the study. The Institutional Ethical Review Boards of Carlos III Institute of Health and *Princesa* Hospital, Madrid, Spain, approved ELEP study.

### Data collection

During the ELEP study course (2007–2010), data was collected yearly during four consecutive years. Symptoms information was gathered once a year while costs were documented during a 3-month period per year. Data included medical care (physicians visits and supportive care visits; hospital admissions; admissions to rehabilitation institutions and diagnostic procedures), PD related drugs (anti-parkinsonian, antidepressants, anxiolytics, hypnotics, antipsychotic drugs), home medical equipment, complementary/alternative medicine and social services/home assistance as well as employment status information.

Based on resource use and productivity losses, direct and indirect costs were calculated for each year, as the mean 3-month costs per patient. Medical resource unit costs were obtained from the Spanish medical cost database Oblikue [15], pharmacy costs from the Spanish college of Pharmaceutics database (Bot Plus Web) [16] and the National Health System official tariffs lists [17]. Estimates of productivity loss due to sick leave, early retirement and medical visits absences were calculated based on one working day pay established by Spanish 2012 annual minimum inter-professional wage [18]. All costs were updated to Euros, October 2012, since statistical analysis were performed by that date.

Taking into consideration the available data on use of concrete medical resources, mean costs were estimated for each individual patient. No methods for handling missing data were applied to these data. For this reason, mean individual cost estimates for the entire population were based on variable sample sizes throughout the analysis.

#### Costs data

Direct medical cost included medical assistance, pharmacological expenses and home medical equipment. Medical assistance cost was calculated multiplying the number of medical and paramedical visits, number of emergency room visits, number and duration of admissions, and number of diagnostic procedures performed during 3 months by the respective unit cost [15]. Pharmacological expenses costs, comprised anti-parkinsonian drugs and other drugs related to PD, were estimated multiplying the cost of daily medication [16] by 90 days (3 months), assuming that patients used the same medication along the study period each year. Home medical equipment covered by the Spanish National Health System was the only proportion of costs considered for the analysis.

Direct non-medical cost included alternative care, home assistance, drugs excluded from the Spanish National Health System and patients’ co-payment for special home medical equipment. Alternative care incorporated cost associated to complementary and alternative medicine. It was calculated multiplying the number of visits performed during the 3-month period by the respective unit cost. Home assistance included costs for domestic help,paid by patients or covered by the Spanish National Health System. They were assessed by multiplying the number of weekly hours of home help by the cost per hour of a home help assistant [17] by 12 weeks, assuming that the patient required domestic help with the same regularity along the observation period. Drugs excluded from the Spanish National Health System included drugs not listed in the national reimbursement drug list and over the counter drugs (OTC) related to PD. This cost was estimated by multiplying the cost of daily medication [15] by 90 days, assuming that patients used the same medication in the same administration basis over the three-month period each year.

Indirect costs related to patient’s productivity loss included sick leave, early retirement and work absences due to medical visits. Costs related to unemployment funds were not considered. Sick leaves involved the number of working days that the patient lost due to PD. It was calculated multiplying the number of days over the 3-month period by the cost of one working day [18]. Early retirement covered productivity loss caused by premature retirement. It was calculated only for patients younger than 65 years old (official retirement age) at the time of study entry multiplying the monthly minimum inter-professional wage [18] by 3 months. To calculate the productivity loss due to medical visits, only employed patients were considered. The number of medical visits reported was multiplied by the cost of half working day (4 hours) [18], assuming that, for attending a medical visit, each patient needed up to 4 hours.

Indirect cost related to informal care included informal care, patient personal care, domestic help and caregiver productivity loss due to medical visits. Informal care was defined as unpaid help provided by family members or friends. To estimate these costs, the replacement valuation method was used (cost of buying a similar amount of services from the formal care sector) [19]. Patient’s personal care cost was assessed by multiplying the number of weekly hours of patient’s care spend by caregiver by 12 (3 months) and by the minimum cost of one hour of nursing assistant, based on the official tariff list [17], assuming that a patient required regular personal care over the period of observation. Since there was no confirmation that the payments made to caregivers were the same as those in the official tariff lists and to avoid cost overestimation, the minimum cost of nursing assistant was used [19]. Cost of domestic help was established multiplying the number of weekly hours spent by the caregiver in domestic help by the minimum cost of a domestic help assistant based on the official tariff list [17], and multiplying it by 12 (3-month). To calculate the productivity loss for caregivers, it was assumed that caregivers spent 4 hours to take PD patients to medical visits. The caregiver’s productivity loss was estimated by multiplying the number of patients’ medical visits reported by 4 hours and by the cost of half working day of a domestic help assistant, based on the official tariff list [17].

#### Clinical data

In addition, clinical and follow up information was also collected by a complete medical and neurological examination performed yearly during the duration of the study. The severity of the disease was measured using the Hoehn and Yahr (HY) staging, which combines motor signs distribution, disability degree, and mobility deterioration. This tool clinically classifies patients into six possible stages from 0 (no signs of disease) to V (needing a wheelchair or bedridden unless assisted), and merges it into 3 categories, mild (HY I and II), moderate (HY III) and severe (HY IV and V) [20].

The SCales for Outcomes in PArkinson's disease–Motor (SCOPA-Motor) is a 21-item instrument, divided in three subscales: motor examination (10-item), activities of daily living (ADL) (7-item) and motor complications (4-item). Item scoring ranges from 0 (normal) to 3 (severe) and a total score from 0 to 75, because four items are scored bilaterally.

Pain was measured using two visual analog scales (VAS), one for intensity and one for frequency of the pain. Both VAS were self-administered by the patient. Scoring ranges from 0 (absence of pain) to 100 (maximum intensity/frequency possible). Total pain score was obtained by multiplying intensity by frequency individual results and dividing it by 100. A VAS scale measured fatigue, which scores ranges from 0 (absence of fatigue) to 100 (worse fatigue possible).

Hospital Anxiety and Depression Scale (HADS) contains 14 items grouped into two dimensions, anxiety and depression. Each item score ranges from 0 (no problem) to 3 (severe problem). The maximum score is 21 for each dimension, being 7 the score above which depression or anxiety are identified. Psychiatric complications were measured using the Parkinson’s Psychiatric Rating Scale (PPRS), a scale designed for the assessment of specific symptoms related to levodopa induced psychosis severity in PD patients. It accounts for 6-item scoring from 1 (without symptoms) to 4 (extreme symptoms), being 6–24 the total possible score range.

SCOPA–Psychosocial (SCOPA-PS) is an 11-item specific self-assessment to measure the psychosocial impact of PD. Item scores range from 0 (not at all) to 3 (very much), providing a summary index by transforming the total sum to a percentage of the maximum possible score (33-point). SCOPA–Sleep is made of two subscales. The nocturnal sleep subscale includes 5-items scoring from 0 (not at all) to 3 (a lot), being 15 the maximum total score. The daytime sleepiness subscale includes 6-items which scores ranges from 0 (never) to 3 (often) being the maximum score 18. In addition, a question regarding overall quality of nocturnal sleep, scoring from 1 (very well) to 7 (very badly) is included.

SCOPA–AUTonomic (SCOPA-AUT) is a 23-item scale designed to assess autonomic dysfunction in different areas: gastrointestinal (7-item), urinary (6-item), cardiovascular (3-item), thermoregulatory dysfunction (4-item), pupillo-motor dysfunction (1-item) and sexual dysfunction (2-item for men and 2-item for women). Each item scores ranges from 0 (never) to 3 (frequently) giving a total score range from 0 to 69. Scales for Outcomes in Parkinson’s disease-cognitive (SCOPA-COG), used to evaluate the cognitive status of Parkinson’s patients, includes 10-item grouped into four domains, namely, attention, memory and learning, executive functions and vision-spatial functions. The total score ranges from 0 to 43, higher score reflect better cognitive performance.

The EQ-5D is a generic HRQoL measure that specifically addresses health status through three main components: descriptive system formed by 5-item, scoring from 1 (no problems or symptoms) to 3 (serious problems or symptoms), a question about change in health status in the preceding 12-month, scoring from 1 (better) to 3 (worse), and a visual analog scale (EQ-VAS) to assess current health status, scoring from 0 (worse imaginable) to 100 (best imaginable). Score profiles for the descriptive part can be converted into an index ranging from 0 (death) to 1 (perfect health), with negative values indicating states worse than death.

In case of missing data in one scale/subscale, it was substituted by the average of answered items in the same scale/subscale provided that the total lost information did not surpass 25%. If the amount of unanswered items in one scale outstripped 25% of the total of items, the scale results were considered as missing data.

### Data Analysis

Descriptive analysis were carried out for all variables, including mean and standard deviation (SD) and range (min-max), and median and range calculations for quantitative variables, and percentage for qualitative ones. With the goal of linking together the clinical evaluations with the 4-year generated costs, linear mixed models were performed analyzing the total, direct and indirect costs based on symptoms, and HRQoL, measured with the previously described scales, using Software R [21] and the statistical package *nlm*e [22]. Total, direct and indirect costs were considered dependent variables and symptoms (motor, non-motor) and HRQoL measured with SCOPA-Motor, pain VAS, fatigue VAS, HADS, PPRS, SCOPA-PS, SCOPA–Sleep, SCOPA-AUT, SCOPA-COG and EQ-5D, as explanatory ones. Only questionnaires with at least 75% of used scales information as well as costs and use of resources of the 4 years of study were included.

A linear mixed model is a parametric linear model for clustered, longitudinal or repeated measures data that qualifies the relationships between a continuous dependent variable and various predictor variables. It provides a linear function of a group of co-variables that estimate the dependent variable and its dependence over time as a fixed effect. Unlike the classic linear regressions, mixed models accounts for repeated observations on each subject over time, because it allows a wide variety of correlation patterns to be explicitly modeled [22].

An important fact to be considered in any statistical regression model is the elimination of observations that, for some unknown reason, disagree with the others and may exert a great influence on the model. To do so, residuals were used to detect deviations from the fitted model against the observed. Raw residuals are defined as the difference between the observed values and the ones predicted by the model. From these raw residuals, other residuals can be defined, the standardized or Pearson residuals (raw residuals divided by the corresponding standard errors). The R functions included in the *nlme* package provided diagnostic plots, using Pearson residuals. Observations with high residuals can distort the results and the accuracy of the regression, so, those observations were removed from the data set and substituted by the sample mean.

## Results

### Patient’s demographics and clinical characteristics

One hundred and seventy four patients from the ELEP cohort were included in the analysis. Half of patients were male (50%). At time of inclusion, the participant’s mean (SD) age was 63 (11) and the mean (SD) disease duration was 8 years (6). The majority of patients had primary education, were married, and lived in urban areas at home and did not belonged to any PD patients association during the follow up period. More than 90% of the participants did not undergo surgery for their Parkinson condition. In those subjects who received a surgical procedure, bilateral sub-thalamic stimulation was the most common one (6.3%). Most patients (83.2%) were inactive workers. The number of patients requiring early retirement increased from year 1 (28.9%) to year 4 (33.9%). The proportion of patients with recognized PD-related disability (disability status provided by the Social Services Ministry) increased 17% during the study period (Table 1).

**Table 1:** Patient’s demographics.

**Mean age, year** *(SD)	63 (11)			
**Mean Parkinson’s duration, year** *(SD)	8 (6)			
**Sex** (%)				
Female	87 (50)			
Male	87 (50)			
**Educational level** (%)				
Primary education	15 (60)			
Secondary education or higher	10 (40)			
	**Year 1**	**Year 2**	**Year 3**	**Year 4**
**Civil status** (%)				
Married	141 (81.0)	135 (80.4)	138 (79.3)	136 (78.2)
Single	16 (9.2)	16 (9.5)	16 (9.2)	16 (9.2)
Widowed	13 (7.5)	14 (8.3)	14 (8.0)	15 (8.6)
Divorced	4 (2.3)	3 (1.8)	6 (3.5)	7 (4.0)
**Place of residence** (%)				
Urban	143 (82.2)	138 (82.1)	147 (84.5)	146 (83.9)
Semi urban	9 (5.2)	9 (5.4)	8 (4.6)	12 (6.9)
Rural	22 (12.6)	21 (12.5)	19 (10.9)	16 (9.2)
**Housing** (%)				
Institutionalized	2 (1.2)	2 (1.2)	8 (4.6)	5 (2.9)
Own home	170 (97.7)	163 (97.0)	164 (94.2)	167 (95.9)
Other	2 (1.2)	3 (1.8)	2 (1.2)	2 (1.2)
**Driving habits** (%)				
No	98 (57.0)	99 (58.9)	106 (61.6)	114 (%)
Yes	74 (43.0)	69 (41.1)	66 (38.4)	59 (34.1)
**Belonging to Patient’s association** (%)				
No	122 (70.1)	112 (67.1)	106 (60.9)	107 (61.8)
Yes	52 (29.9)	55 (32.9)	68 (39.1)	66 (38.2)
**Surgeries related to Parkinson** (%)				
Without surgery	161 (92.5)	160 (100)	156 (98.1)	150 (98.0)
Unilateral sub thalamic stimulation	1 (0.6)	0	1 (0.6)	3 (2.0)
Bilateral sub thalamic stimulation	11 (6.3)	0	2 (1.3)	0
Other unilateral surgery	1 (0.6)	0	0	0
**Employment status** (%)				
Active worker	29 (16.8)	22 (12.6)	17 (9.8)	14 (8.0)
Inactive worker	144 (83.2)	152 (87.4)	157 (90.2)	160 (92.0)
Earlier retired due to Parkinson’s	50 (28.9)	55 (31.6)	57 (32.8)	59 (33.9)
Sick leave due to Parkinson’s	0	0	3 (1.7)	2 (1.1)
Unemployed	0	1 (0.6)	1 (0.6)	2 (1.1)
Illness or disability pensioner	0	32 (18.4)	40 (23.0)	44 (25.3)
Housewife	32 (18.5)	37 (21.3)	34 (19.5)	33 (19.0)
**Disability Status** (%)				
Without disability	64.7	58.7	49.4	48.0
1–25%	0.1	0.5	0.5	0.5
26–50%	8.8	7.0	12.4	10.4
51–75%	12.9	16.9	17.1	21.4
76–100%	13.5	16.9	20.6	18.5
Great disability >150%	0	0	0	1.2

*At inclusion, SD: standard deviation

### Clinical and follow up results

**Fig 1:**
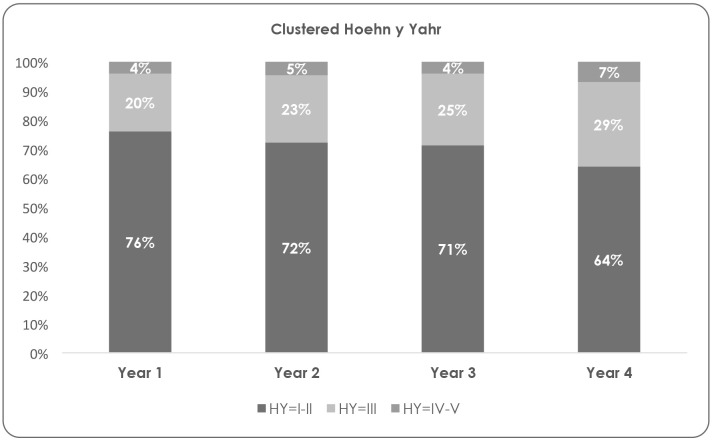
Patients distribution by disease severity according to clustered Hoehn and Yahr staging.

More than 95% of participants were classified as HY stage I, II or III (mild or moderate disease). Forty-nine percent (n = 85) of patients remained in the same disease stage during the four years of study, while 34% (n = 59) progressed one stage, 6% (n = 10) two stages and only 1% (n = 1) advanced three stages between year 1 and 4 (Fig 1).

SCOPA-Motor results evidenced a moderate change between the first [mean (SD) year 1: 39.3 (8.4)] and the last evaluation [mean (SD) year 4: 42.5 (9.5)]. Similar findings emerged with the SCOPA-COG result [mean (SD) year 1: 24.9 (6.2)]; year 4: 24.1 (7.4)] and SCOPA-AUT [mean (SD) year 1: 20.3 (11.3); year 4: 22.0 (10.1)]. SCOPA-PS results demonstrated a mild increment of the disease impact on patients’ psychosocial adjustment over time [mean (SD) year 1: 6.9 (5.7); year 4: 8.2 (5.8)].

PPRS scores [mean (SD) year 1: 1.0 (1.3); year 4: 1.2 (1.5)] indicates that most participants did not evidenced psychiatric complication, such as hallucinations or confusion throughout the four years of study. In regards to HADS, study subjects presented scores above 7 in the anxiety dimension [mean (SD) year 1: 7.3 (4.1); year 4: 7.1 (4.3)] but not in the depression one [mean (SD) year 1: 5.4 (3.5); year 4: 5.9 (3.9)]. According to the SCOPA-Sleep scores, participants did not present alterations in neither daytime [mean (SD) year 1: 3.8 (3.2); year 4: 3.8 (3.0)] nor nocturnal sleep [mean (SD) year 1: 4: 5.5 (4.0); year 4: 4.8 (3.5)] and these results did not vary during the time of study. Pain [mean (SD) year 1: 19.2 (21.4); year 4: 22.9 (23.7)] and fatigue [mean (SD) year 1: 25.3 (27.9); year 4: 33.7 (29.4)] levels increased slightly.

EQ-5D questionnaire results showed that participants had an acceptable HRQOL (EQ-5D index), which remained stable during the study period [mean (SD) year 1: 0.7 (0.3); year 4: 0.6 (0.3)]. On the other hand, EQ-VAS showed a mild reduction of HRQoL as time went by [mean (SD) year 1: 63.7 (20.3); year 4: 60.3 (17.9)].

### Costs of Parkinson disease

As presented in Table 2, the mean total costs of PD increased 92.5% from a mean of €2,082.17 (SD: €2,889.86, n = 174) in year 1 to €4,008.6 (SD: €7,757.35, n = 174) in year 4. Mean direct costs increased 52% from €1,330.51 (SD: €2,229.77, n = 174) in year 1 to €2,022.90 (SD: €4,582.78, n = 166) in year 4. Mean direct medical costs per patient rose 52.7% over time, from €998.38 (SD: €1,476.15, n = 174) at year 1 to €1,525.00 (SD: 4,321.92, n = 166) in year 4. Pharmacological costs per patient increased 81.6% over time, from €284.39 (SD: 241.87, n = 174) at year 1 to €516.48 (SD: 458.92, n = 174) at year 4. Mean direct non-medical costs, including alternative care and home assistance among others, also showed an increase of 161.38% from €1,090.40 (SD: €2,781.15, n = 53) in the first year up to €2,850.10 (SD: €2,839.70, n = 29) in the fourth year. In regards of indirect costs, less than 53% of patients had registered data in the case report form (CRF). The mean indirect cost, comprehending productivity loss and informal care, heightened 128.45% from €1,720.90 (SD: €2,176.90, n = 76) in the first year to €3,931.40 (SD: €8,372.62, n = 92) in the fourth year.

**Table 2:** Description of Parkinson’s disease costs during the four years of study.

	Year 1				Year 2				Year 3				Year 4			
	Mean €	SD €	Range €	n	Mean	SD	Range	n	Mean	SD	Range	n	Mean	SD	Range	n
**Direct costs**	**1,330.51**	**2,229.77**	**16.65–20,948.08**	**174**	**1,088.20**	**1,966.87**	**2.03–22,298.30**	**174**	**1,819.49**	**3,008.92**	**25.2–22,620.09**	**174**	**2,022.90**	**4,582.78**	**25.37–51,180.60**	**166**
Medical	998.38	1,476.15	8.10–10,442.78	174	763.66	729.10	2.03–6,461.14	174	914.34	1,027.60	12.15–9,261.79	174	1,525.00	4,321.92	25.37–51,180.60	166
Pharmacological	284.39	241.87	0.00–1220.07	174	393.76	355.21	0.00–3132.94	174	466.00	347.89	5.40–1602.88	174	516.48	458.92	0.00–3807.27	174
Non-medical	1,090.40	2,781.15	6.00–19,857.60	53	1,660.88	3,853.10	6.30–22,063.80	34	4,144.66	4,715.82	15.00–20,157.66	38	2,850.10	2,839.70	20.00–8,903.56	29
**Indirect costs**	**1,720.90**	**2,176.90**	**21.38–12,830.48**	**76**	**1,865.30**	**4,916.66**	**21.38–44,812.88**	**85**	**2,062.48**	**4,947.42**	**21.38–44,775.36**	**93**	**3,931.40**	**8,372.62**	**37.52–55,680.62**	**92**
**Total Costs**	**2,082.17**	**2,889.86**	**16.65–20,948.08**	**174**	**1,999.41**	**4,199.18**	**2.03–46,631.75**	**174**	**2,921.85**	**5,998.40**	**25.20–67,395.45**	**174**	**4,008.60**	**7,757.35**	**37.52–56,730.81**	**174**

SD: standard deviation

**Fig 2:**
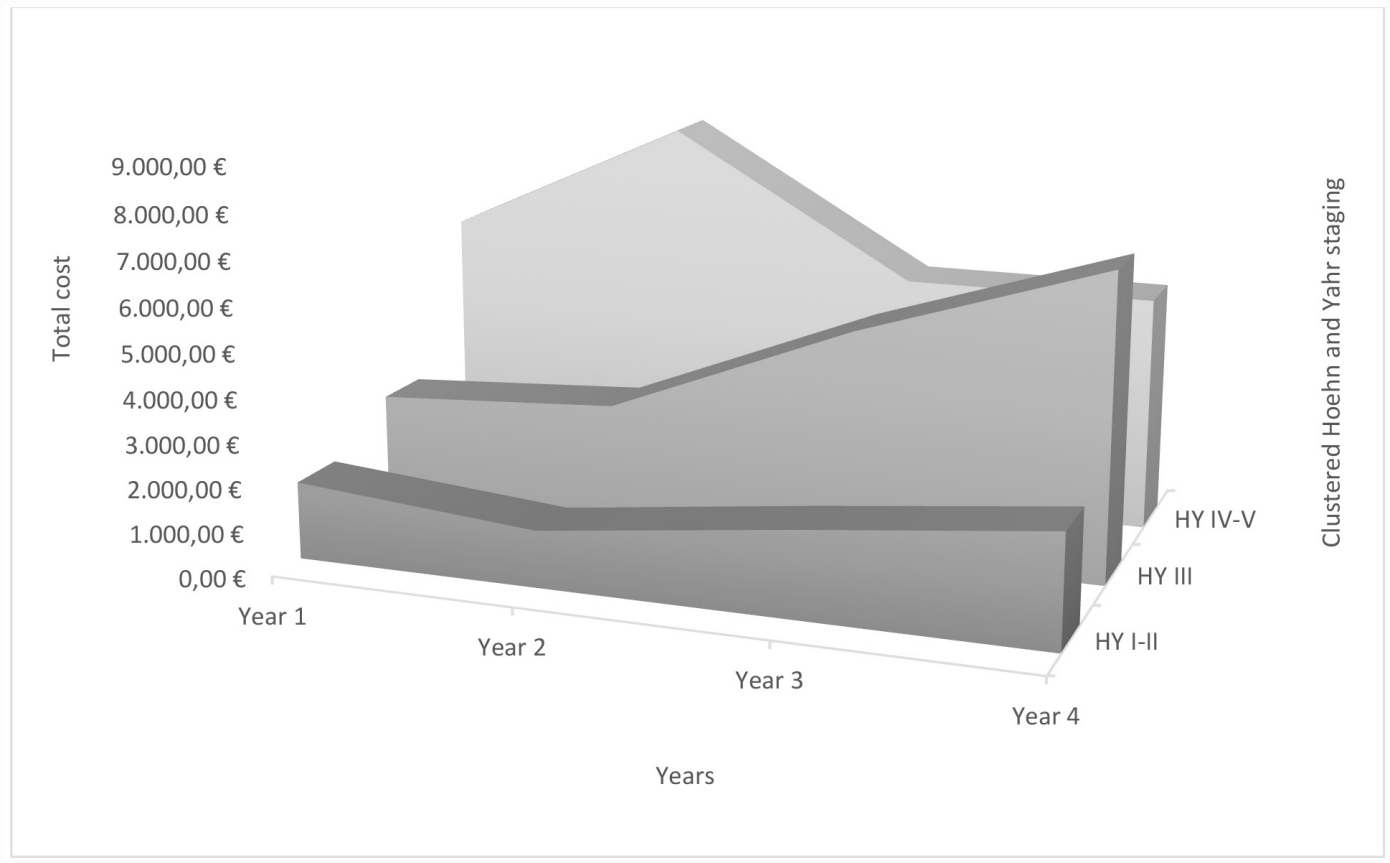
Total costs due to disease progression—disease severity according to clustered Hoehn and Yahr staging.

When analyzing PD costs variation by disease severity over the 4 years of study, mean total, direct and indirect costs were always higher at year 4 for the mild and moderate groups (Fig 2). Total costs, direct costs and indirect cost were 45.96%, 35.63% and 69.69% higher in mild disease, respectively, whereas there was an increment of 166.52% for total costs, 55.68% for direct cost and 347.85% for indirect cost in moderate PD population. In severe patients, cost remained almost the same during the study period. All costs are presented in Table 3.

**Table 3:** Description of Parkinson’s disease costs during the four years of study by disease severity.

	Year 1			Year 2			Year 3			Year 4		
	Direct Cost	Indirect Cost	Total Cost	Direct Cost	Indirect Cost	Total Cost	Direct Cost	Indirect Cost	Total Cost	Direct Cost	Indirect Cost	Total Cost
**HY I-II**												
**N**	131	52	131	125	49	125	122	53	122	108	43	110
**Mean**	**1,089.29**	**1,659.78**	**1,748.14**	**773.24**	**1,291.21**	**1,279.39**	**1,337.09**	**1,395.22**	**1,943.21**	**1,477.43**	**2,816.47**	**2,551.55**
**SD**	1,614.17	2,000.76	2,411.94	903.71	1,562.13	1,569.83	1,817.52	1,950.19	2,448.41	2,309.24	3,004.36	3,395.28
**Minimum**	16.65	21.38	16.65	2.03	21.38	2.03	25.20	21.38	25.20	30.60	37.52	37.52
**Maximum**	10,442.78	10,777.20	13,716.06	6,540.90	9,328.20	9,665.63	10,774.41	12,932.64	14,397.49	18,198.36	16,662.21	20,680.56
**HY III**												
**N**	34	16	34	40	27	40	42	30	42	49	35	50
**Mean**	**2,017.71**	**1,222.59**	**2,593.05**	**1,949.78**	**1,416.39**	**2,905.84**	**2,920.56**	**3,079.85**	**5,120.45**	**3,141.10**	**5,475.38**	**6,911.04**
**SD**	3,622.67	1,153.44	3,671.23	3,650.10	1,252.08	4,050.30	4,926.04	8,067.23	10,870.59	7,599.18	12,611.05	12,698.30
**Minimum**	179.94	37.50	179.94	36.00	37.52	52.20	76.50	37.52	114.02	25.37	37.52	37.52
**Maximum**	20,948.08	3,570.70	20,948.08	22,298.34	4,092.24	24,233.23	22,620.09	44,775.36	67,395.45	51,180.60	55,680.62	56,730.81
**HY IV-V**												
**N**	7	7	7	8	8	8	7	7	7	7	12	12
**Mean**	**2,656.27**	**3,281.81**	**5,938.09**	**1,616.21**	**6,881.98**	**8,498.18**	**2,342.99**	**2,977.15**	**5,320.14**	**2,682.55**	**3,745.42**	**5,310.24**
**SD**	2,929.58	4,282.39	4,325.54	716.79	15,351.78	15,429.76	1,899.33	4,120.90	5,849.04	2,076.70	6,328.80	7,176.07
**Minimum**	489.78	75.04	1,476.00	559.71	37.52	2,120.19	698.91	37.52	2,064.79	315.00	37.52	37.52
**Maximum**	8,369.29	12,830.48	13,397.97	2,450.50	44,812.88	46,631.75	6,402.87	12,083.92	18,486.79	6,673.52	19,879.60	21,545.95

HY: Hoehn and Yahr; SD: standard deviation

Mean total costs for PD were 48.33% and 239.68% higher than mild PD for moderate and severe disease during year 1 and 170.86% and 108.12% higher for moderate and severe disease during year 4, respectively. For the first year mean total cost for mild disease was €1,748.14 (SD: €2,411.94, n = 131), €2,593.05 (SD: €3,671.23, n = 34) for moderate and €5,938.09 (SD: €4,325.54, n = 7) for severe PD. During year 4, mean total cost for PD was €2,551.55 (SD: €3,395.28, n = 110) for mild, €6,911.04 (SD: 12,698.30, n = 50) for moderate and €5,310.24 (SD: €7,176.07, n = 12) for severe PD.

Mean direct costs for PD were 85.23% and 143.85% higher for moderate and severe disease during year 1 and 112.61% and 81.57% higher for moderate and severe disease during year 4, respectively, compared to mild PD. For the first year, mean direct cost for mild disease was €1,089.29 (SD: €1,614.17, n = 131), €2,017.71 (SD: €3,622.67, n = 34) for moderate and €2,656.27 (SD: €2,929.58, n = 7) for severe disease. During year 4, mean direct cost for PD was €1,477.43 (SD: €2,309.24, n = 108) for mild, €3,141.10 (SD: €7,599.18, n = 49) for moderate and €2,682.55 (SD: €2,076.70, n = 7) for severe PD.

Compared to mild disease, mean indirect cost for PD were 26.34% lower and 97.73% higher for moderate and severe disease during year 1 and 94.41% and 32.98% higher for severe disease during year 4, respectively. For the first year, mean indirect cost for mild disease was €1,659.78 (SD: €2,411.94, n = 52), €1,222.59 (SD: €1,153.44, n = 16) for moderate and €3,281.81 (SD: €4,282.39, n = 7) for severe disease. During year 4, mean indirect cost for PD was €2,816.47 (SD: €3,004.36, n = 43) for mild, €5,475.38 (SD: €12,611.05, n = 35) for moderate and €3,745.42 (SD: €6,328.80, n = 12) for severe disease.

When disease motor symptoms, non-motor symptoms and HRQoL were modeled against total costs, results showed that for each point increment in the SCOPA-Motor scale total costs augmented €75.72 per patient(p = 0.0174) (Table 4). The same analysis with direct costs demonstrated that for each additional point in the SCOPA-Motor and the SCOPA-COG scale, direct costs incremented in €49.21 (p = 0.0094) and €44.81 per patient (p = 0.0404), respectively. However, higher scores in HADS-D scale associated with savings of €133.24 per patient (p = 0.079). When the model included indirect costs, pain was the only symptom that influenced it, being that for each additional point on the pain VAS scale, indirect costs heightened €16.31 per patient (p = 0.0228).

**Table 4:** Linear mixed model of cost based on symptoms and HRQoL.

**Linear mixed model of total cost based on symptoms and HRQoL**		
**Response variable: Total cost**	**Estimated Coefficient**	**p-value**
Constant	-416.0789	0.7844
SCOPA Motor	75.7167	0.0174
**Linear mixed model of direct cost based on symptoms and HRQoL**		
**Response variable: Direct cost**	**Estimated Coefficient**	**p-value**
Constant	341.3715	0.7029
SCOPA Motor	49.21	0.0094
HADS D	-133.2406	0.0079
SCOPA COG	44.8088	0.0404
**Linear mixed model of indirect cost based symptoms and HRQoL**		
**Response variable: Indirect cost**	**Estimated Coefficient**	**p-value**
Constant	-517.7612	0.6038
Pain	16.3129	0.0228

SCOPA Motor: Scales for outcomes in Parkinson disease—Motor; HADS A: Hospital anxiety and depression scale—anxiety; HADS D: Hospital anxiety and depression scale—depression; PPRS: Parkinson’s psychiatric rating scale; SCOPA PS: Scales of outcomes in Parkinson’s disease—Psychosocial; SCOPA Sleep: Scales for outcomes in Parkinson’s disease—Sleep; SCOPA AUT: Scales of outcomes in Parkinson’s disease—Autonomic; SCOPA COG: Scales of outcomes in Parkinson’s disease—Cognitive; EQ-VAS: EuroQoL Visual analogue scale.

## Discussion

This study provides the first opportunity for the longitudinal examination of the clinical and socioeconomic impact of Parkinson in the Spanish population. There are three main aspects derived from this analysis: the study population was a well-controlled one with a moderate disease that had minimal change over the study period, associated costs markedly increased over time and motor symptoms, cognitive dysfunction and pain had a direct impact on disease expenditure.

Albeit the inclusion criteria used for the selection of the sample in the ELEP study, in which sociodemographic and clinical variables were considered in order to avoid typical biases (inclusion based on severity levels according to HY criteria, which provides asymmetrical samples with overrepresentation of HYII and HYIII and practical absence of HYV), in this study the majority of the sample had PD HYI-III. These results concurred with the epidemiology data of Parkinson in Spain, which states that more than 60% of Parkinson patients are HY I-II and around 25%, HY IV-V [10]. In addition, patient’s condition remained stable during the study period. Most patients presented intermediate scores in the symptoms assessments with mild variations over time. Moderate scores were also consistently observed for HRQoL evaluation. These can be explained because specialists who were experts in managing PD and controlling the disease over time followed the included subjects.

Results in the present publication demonstrates that PD total cost per patient in Spain increased over time, from €2,082.17 per trimester in year 1 to €4,008.60 per trimester in year 4. These results are in line with previous publications. Garcia Ramos et al. (2013) [10] reviewed all published data regarding PD costs in Spain and reported total costs of €8,640 per semester, being direct costs the biggest contributor to this amount reaching €6,030, while Von Campenhausen et al. (2011) [23], demonstrated that PD total costs of per semester, from the societal perspective, were lower in Eastern European countries (Russia: €2,620, Czech Republic: €5,510) as compared with most Western European countries (Austria: €9,820, Germany: € 8,610, Italy: €8,340). Moreover, Schenkman et al. (2001) [24] affirmed that costs augmented mainly because of the temporal progression of the disease.

Data presented in this publication also reveals that disease progression is a key driver on disease costs, being that total, direct and indirect cost increases as HY states do. This finding has also been described by other authors. Cubo et al. (2009) [25] in their cross sectional pilot study in Spain during 2004 reported that direct cost were significantly higher in patients with severe disease (p<0.001) and longer disease duration (p<0.01). Findley (2007) [26] in their review of the economic impact of PD in UK also stated that disease severity was the single most important cost driver. Their data showed that patients diagnosed with severe Parkinson (HY V) incurred in costs that were up to six times higher than patients with mild disease (HY I) (€3 583,08 vs. €22176.46). Lastly, Keränen et al. (2003) [11] in their observational study with Finnish Parkinson patients, concluded that there was an association between disease severity and annual costs, showing that the proportion of indirect costs increased as the disease progressed, accounting for as much as half of the total costs in severe (HY IV) stages.

Regarding PD symptoms, the presented analysis demonstrates that motor symptoms significantly affected PD total and direct costs. Considering that resting tremor, bradykinesia, rigidity and postural instability are the main motor complications of PD and have a prevalence of up to 90% [27], this finding results paramount. Concurring with this statement, Reese et al. (2011) [28] in their study with PD patients assessed the clinical condition, disease severity and related it to total costs, reporting that motor fluctuations were the main total costs predictor, being total cost for patients with motor fluctuations 113.8% higher than patients without motor fluctuations. In the same line, Cubo et al. (2009) [25] in their study relating motor and non-motor symptoms with direct costs, also made a significant correlation between motor symptoms measured by SCOPA-Motor and direct costs.

In addition to the later, this publication also shows that cognitive impairment is another significant predictor of direct costs. In agreement with this, Vossius et al. (2011) [29] performed a standard regression analysis to evaluate the effect of dementia on direct costs per year of survival (YOS) in Norwegian PD patients, indicating that direct costs of patients with dementia were 3.3 times higher than those without dementia per YOS in 1997. In addition, institutional care was the largest cost factor, representing 67% of direct costs, cognitive function predicted direct costs in 29.4% and cognitive decline was associated with increased costs, even in non-demented patients.

In this study depression resulted inversely related to PD direct costs, being that patients with depression had fewer expenses. This finding was consistent with other published article that demonstrated that PD patients with depression expend less in treatments decreasing pharmacological costs [30]. In line with this statement, Richy et al. (2013) [31] in their analysis of compliance with pharmacotherapy and direct costs in subjects with PD demonstrated that depressed patients were at increase odds to be non-compliant compared to patients without this diagnose (p<0.0001). Non-compliant patients expended 38.8% less in pharmacologic treatments than compliant ones. Despite the later, this data should be carefully considered since it only considers pharmaceutical cost disregarding the use of resources that can derive from it. More studies should be performed on this subject.

The main goal of treatment guidelines for motor and non-motor symptoms are to preserve functional independence and HRQoL. Achievement of these objectives can reduce the need for healthcare resources utilization and decrease associated costs. Treatment should be directed at providing symptomatic relief for motor and non-motor symptoms with special interest in cognitive impairment and pain, while minimizing undue adverse effects [32].

Finally, another important finding provided by this study is that pain is associated with an increment in indirect costs. One can argue that PD has not been clearly related with pain, however it is present in around 40% of PD patients [33]. Moreover, chronic pain also markedly decrease HRQoL and health status of PD patients [34] and can relate with depression (OR = 2.88, 95%CI: 1.87–4.44) [35]. The relation of pain and indirect cost increments has been previously denoted across Europe. Almost one in five patients with chronic pain reported having lost their jobs because of their pain and one third reported that the hours they worked or whether they worked at all, were affected by their pain, which is associated with an enormous indirect cost that can represent 3–10% of the gross domestic product. Among the types of pain, neuropathies appeared among the most costly ones. Productivity loses, absenteeism and early retirement and disability retirement contributed substantially to these costs, and could be reduced by investing in effective therapeutic interventions [34]. The clinical approach for the vast majority of PD related pain is the optimization of levodopa treatment [36].

This study has limitations due to the population included. The majority of patients had mild to moderate PD, which remained stable over time hindering the evaluation of severe disease groups, who are probably the ones who need more resources and are most costly to the system. Moreover, unlike Schenkman et al. (2001) study [24], which was created with the objective of assessing economic impact of PD over 3 years, the ELEP study was not designed for costs estimations; therefore, cost information was only registered over a three-month period assuming patients used the same resources over that time. Finally, considering that only descriptive analysis were carried out, further research including clinical sample differences should be conducted.

## Conclusions

PD causes an important economic impact for the Spanish National Health System. Considering that the presented results included patients with mild to moderate disease, it can be suggested that severely ill patients would represent a bigger economic burden for the system.

Total costs increased with the progression and the severity of the disease, being motor symptoms, cognitive impairment and pain main predictors of costs. Therapeutic measures aimed at slowing disease progression and controlling of motor and cognitive symptoms, as well as pain, could help to contain disease expenditures, although further global analysis is required in order to obtain more definitive conclusions.

## Supporting Information

S1 File Dataset used for the analysis. (CSV)

## References

[1] Chen JJ. Parkinson’s disease: health related quality of life, economic cost, and implications of early treatment. Am J Manag Care. 2010;16:S87–S93. 20297871

[2] Dorsey ER, Constantinescu R, Thompson JP, Biglan KM, Holloway RG, Kieburtz K, et al Projected number of people with Parkinson’s disease in the most populous nations, 2005 through 2030. Neurology. 2007;68(5):384–386. 17082464 10.1212/01.wnl.0000247740.47667.03

[3] Peñas Domingo E, Gálvez Sierra M, Marín Valero M, Pérez-Olivares Castiñeira M. Libro Blanco del Parkinson en España. Real Patronato sobre Discapacidad (Ministerio de Sanidad, Servicios Sociales e Igualdad). 2015. Available at: http://www.fedesparkinson.org/libro_blanco.pdf. Accessed: July 2015.

[4] Bloem BR, Stocchi F. Move for Change Part I: a European survey evaluating the impact f teh EPDA Charter for People with Parkinson’s disease. European Journal of Neurology. 2012;19:402–410. 10.1111/j.1468-1331.2011.03532.x 21967281 PMC3489042

[5] Gustavsson A, Svensson M, Jacobi F, Allgulander C, Alonso J, Beghi E, et al Cost of disorders of the brain in Europe 2010. European Neuropsychopharmacology. 2011;21:718–779. 10.1016/j.euroneuro.2011.08.008 21924589

[6] Winter Y, Balzer-Geldsetzer M, von Campenhausen S, Spottke A, Eggert K, Oertel W, et al Trends in resource utilization for Parkinson’s disease in Germany. Journal of the Neurological Sciences. 2010;294(1–2):18–22. 10.1016/j.jns.2010.04.011 20493500

[7] Findley LJ. The economic impact of Parkinson’s disease. Parkinsonism & Related Disorders. 2007;13(Suppl.):S8–S12. 17702630 10.1016/j.parkreldis.2007.06.003

[8] Mateus C, Coloma J. Heath economics and cost of illness in Parkinson’s disease. European Neurological Review. 2013;8(1):6–9.

[9] Dodel R. Interpreting Health Economics Data in Parkinson’s disease. European Neurological Review. 2011;6(Suppl.1):13–16.

[10] García Ramos R, López Valdez E, Ballesteros L, Jesus S, Mir P. Informe de la fundación del cerebro sobre el impacto social de la enfermedad de Parkinson en España. Neurología. 2013 10.1016/j.nrl.2013.04.008 23816428

[11] Keränen T, Kaakkola S, Sotaniemi K, Laulumaa V, Haapaniemi T, Jolma T, et al Economic burden and quality of life impairment increase with severity of PD. Parkinsonism Relat Disord. 2003 9:163–168. 12573872 10.1016/s1353-8020(02)00097-4

[12] Boland D, Stacy M. The economic and quality of life burden associated with Parkinson’s disease: a focus on symptoms. Am J Manag Care. 2012;18(7):S168–S175. 23039865

[13] ELEP group. Estudio longitudinal de pacientes con enfermedad de Parkinson (ELEP): objetivos y metodología. Rev Neurol. 2006;42 (6):360–365. 16575773

[14] Hughes AJ, Daniel SE, Kilford L, Lees AJ. Accuracy of clinical diagnosis of idiopathic Parkinson’s disease. A clinico-pathological study of 100 cases. JNNP. 1992;55:181–184. 10.1136/jnnp.55.3.181 PMC1014720 1564476

[15] Oblikue consulting. eSalud. Online medical economic database. Acceded: November 2012. Available at: http://oblikue.com/bdcostes/. Accessed July 2013.

[16] Consejo General de Colegios Oficiales de Farmacéuticos. Bot Plus Web. Available at: https://botplusweb.portalfarma.com/. Accessed July 2013.

[17] Boletín Oficial del Estado N° 122, 23 de mayo de 2011. Available at: http://www.boe.es/boe/dias/2011/05/23/pdfs/BOE-S-2011-122.pdf. Accessed July 2013.

[18] Boletín Oficial del Estado N° 314, 31 de diciembre de 2011. Available at: http://www.boe.es/boe/dias/2012/12/31/pdfs/BOE-S-2012-314.pdf. Accessed July 2013.

[19] Olivia J, Osuna R, Jorgensen N. Estimación de los costes de los cuidados informales asociados a enfermedades neurológicas de alta prevalencia en España. Pharmacoeconomics. 2007;4(3):83–96. 18365777

[20] Armañazas R, Bielza C, Chaudhuri KR, Martinez-Martin P, Larrañaga P. Unveiling relevant non-motor Parkinson’s disease severity symptoms using machine learning approach. Artif Intell Med. 2013;58:195–202. 10.1016/j.artmed.2013.04.002 23711400

[21] R Core Team. R: A language and environment for statistical computing. R Foundation for Statistical Computing, Vienna, Austria 2012 ISBN 3-900051-07-0. Available at: URL http://www.R-project.org/. Accessed July 2015.

[22] Pinheiro J, Bates D, DebRoy S, Sarkar D, EISPACK authors. Linear and Nonlinear Mixed Effects Models. R package version 3. 2013:1–113. Available at: https://cran.r-project.org/web/packages/nlme/nlme.pdf. Accessed July 2014.

[23] Von Campenhausen s, Winter Y, Rodriguez e Silva A, Sampaio C, Ruzicka E, Barone P, et al Cost of illness and care in Parkinson’s disease: an evaluation on six countries. Eur Neuropsychopharm. 2011;21:180–191. 10.1016/j.euroneuro.2010.08.002 20888737

[24] Schenkman M, Wei Zhu C, Cutson TM, Whetten Goldstein K. Longitudinal evaluation of economic and physical impact of Parkinson’s disease. Parkinsonism and Related Disorders. 2001;8:41–50. 11472879 10.1016/s1353-8020(00)00079-1

[25] Cubo E, Martinez Martin P, Gonzalez M, Frades B. Impacto de los síntomas motores y no motores en los costes directos de la enfermedad de Parkinson. Neurología. 2009;21(1):15–23. 19003550

[26] Findley LJ. The economic impact of Parkinson’s disease. Parkinsonism Relat Disord. 2007;13: S8–S12. 17702630 10.1016/j.parkreldis.2007.06.003

[27] Weintraub D, Comella CL, Horn S. Parkinson’s disease—Part I: Pathophysiology, symptoms, burden, diagnosis and assessment. Am J Manag Care. 2008;14:S40–S48. 18402507

[28] Reese JP, Winter Y, Rosa MM, Rodriguez e Silva AM, von Campenhausen S, Freire R, et al Estudio de cohortes sobre la carga socioeconómica de la enfermedad de Parkinson en Portugal. Neurologia. 2011;52(5):264–274. 21341221

[29] Vossius C, Larsen JP, Janvin C, Aarsland D. The economic impact of cognitive impairment in Parkinson’s disease. Movement Disord. 2011;26(8):1541–1544. 10.1002/mds.23661 21538519

[30] Bach JP, Riedel O, Klotsche J, Spottke A, Dodel R, et al Impact of complications and comorbidities on treatment cost and Health related quality of life of patients with Parkinson’s disease. J Neurol Sci. 2012;314:41–47. 10.1016/j.jns.2011.11.002 22133477

[31] Richy FF, Pietri G, Moran KA, et al Compliance with pharmacotherapy and direct healthcare costs in patients with Parkinson’s disease: a retrospective claims database analysis. Appl Heath Econ Health Policy. 2013;11:395–406. 10.1007/s40258-013-0033-1 PMC3717155 23649891

[32] Weintraub D, Comella C, Horn S. Parkinson’s disease—Part 2: treatment of motor symptoms. Am J Manag Care. 2008;14:S49–S58. 18402508

[33] Ford B. Pain in Parkinson’s. Movement Disord. 2010;25(S1):S98–S103. 20187254 10.1002/mds.22716

[34] Breivik H, Eisenberg E, O’Brien T. The individual and societal burden of chronic pain in Europe: the case for strategic prioritisation and action to improve knowledge and availability of appropriate care. BMC Public Health. 2013;13:1229 http://biomedcentral.com/1471-2458/13/1229. 10.1186/1471-2458-13-1229 24365383 PMC3878786

[35] Wen HB, Zhang ZX, Wang Hm, Li L, Chen H, Liu Y, et al Epidemiology and clinical phenomenology for Parkinson’s disease with pain and fatigue. Parkinsonism Relat Disord 2012;18 S1:S222–S225. 22166441 10.1016/S1353-8020(11)70068-2 PMC3648843

[36] Chaudhuri KR, Martinez Martin P, Odin P, Antonini A. Handbook of Non Motor symptoms in Parkinson Disease. 2011© Springer Healthcare.

